# Evaluating the Efficacy of Taurodeoxycholic Acid in Providing Otoprotection Using an *in vitro* Model of Electrode Insertion Trauma

**DOI:** 10.3389/fnmol.2020.00113

**Published:** 2020-07-14

**Authors:** Viraj Shah, Rahul Mittal, David Shahal, Priyanka Sinha, Erdogan Bulut, Jeenu Mittal, Adrien A. Eshraghi

**Affiliations:** ^1^Cochlear Implant and Hearing Research Laboratory, Department of Otolaryngology, University of Miami Miller School of Medicine, Miami, FL, United States; ^2^Department of Neurological Surgery, University of Miami Miller School of Medicine, Miami, FL, United States; ^3^Department of Biomedical Engineering, University of Miami, Coral Gables, FL, United States

**Keywords:** cochlear implant, taurodeoxycholic acid, electrode insertion trauma, oxidative stress, caspase-3 pathway, nitric oxide

## Abstract

Cochlear implants (CIs) are widely used to provide auditory rehabilitation to individuals having severe to profound sensorineural hearing loss (SNHL). However, insertion of electrode leads to inner trauma and activation of inflammatory and apoptotic signaling cascades resulting in loss of residual hearing in implanted individuals. Pharmaceutical interventions that can target these signaling cascades hold great potential for preserving residual hearing by preventing sensory cell damage. Bile salts have shown efficacy in various regions of the body as powerful antioxidants and anti-inflammatory agents. However, their efficacy against inner ear trauma has never been explored. The objective of this study was to determine whether taurodeoxycholic acid (TDCA), a bile salt derivative, can prevent sensory cell damage employing an *in vitro* model of electrode insertion trauma (EIT). The organ of Corti (OC) explants were dissected from postnatal day 3 (P-3) rats and placed in serum-free media. Explants were divided into control and experimental groups: (1) untreated controls; (2) EIT; (3) EIT+ TDCA (different concentrations). Hair cell (HC) density, analyses of apoptosis pathway (cleaved caspase 3), levels of reactive oxygen species (ROS) as well as inducible nitric oxide synthase (iNOS) activity and Mitochondrial Membrane Potential (MMP) were assayed. Treatment with TDCA provided significant otoprotection against HC loss in a dose-dependent manner. The molecular mechanisms underlying otoprotection involved decreasing oxidative stress, lowering levels of iNOS, and abrogating generation of cleaved caspase 3. The results of the present study suggest that TDCA provides efficient otoprotection against EIT, *in vitro* and should be explored for developing pharmaceutical interventions to preserve residual hearing post-cochlear implantation.

## Introduction

Sensorineural hearing loss (SNHL) affects one in three Americans in the adult population, and worldwide approximately half a billion people have disabling hearing loss (Müller and Barr-Gillespie, 2015; Wilson et al., 2017). Furthermore, its increasing prevalence has elevated SNHL to be the fourth leading cause of years lived with disability (Wilson et al., 2017). Cochlear implantation is a common procedure performed in individuals with complete deafness or severe to profound hearing loss. Whereas hearing aids work to amplify sound, a cochlear implant (CI) mimics the action of the cochlea by directly stimulating the spiral ganglion neurons in the inner ear and sending signals to the central auditory processing center of the brain which enables the detection of environmental auditory stimuli. Individuals with post-lingual hearing loss benefit from CIs not only in the areas of language and speech, but also in holistic assessments of the quality of life (de Sousa et al., 2018). CIs help in improving speech perception and auditory skills as well as improvements in expressive and receptive language (Carlson et al., 2015; McKinney, 2017; Li Y. et al., 2019; Gagnon et al., 2020). These developments in speech occur as CIs allow children to hear the sounds of speech to stimulate, develop and refine their acoustic and phonologic centers in the brain allowing them to interact with the surrounding environment (Markman et al., 2011; Liu et al., 2019; Guo et al., 2020; Jiang et al., 2020). Various behavioral changes also occur post-cochlear implantation such as improvements in attention span and reduced distractibility which lead to improved cognitive function, communication skills, and overall expressive language skills (Pulsifer et al., 2003). CI technology has improved tremendously over the past 40 years in the areas of electrode array design, CI insertion approaches, and otoprotective drug use as well as most recently in the employment of robotic surgery to access the middle ear (Eshraghi et al., 2012; Caversaccio et al., 2019). Previously unattainable improvements in areas of music appreciation and the ability to discern speech in a loud environment are also becoming plausible (Eshraghi et al., 2012).

The insertion of the electrode array during cochlear implantation can induce trauma of the basilar membrane and the spiral lamina. This trauma can induce an inflammatory response that leads to further damage of inner ear hair cells (HCs) and loss of residual hearing. In particular, electrode array design is important for optimizing functionality with CI as well as limiting potential insertion trauma. The two main types of designs commercially available are the straight lateral wall (LW) electrode arrays and pre-curved modiolar hugging (MH) electrode arrays (Dhanasingh and Jolly, 2017). The straight LW electrode comes in a multitude of lengths that can be fitted according to the size of the individual’s cochlea, whereas the MH electrode array allows for closer positioning to the modiolar wall. Electrode design has evolved significantly over the past several years; however, insertion trauma still poses a significant risk of losing residual hearing post-cochlear implantation. Therefore, while children and individuals can benefit from CIs in natural age-related hearing loss or noise-induced hearing loss, the procedure is not performed due to the risk of potential loss of residual hearing that may be attributed to electrode insertion trauma (EIT). There has been an increased interest in expanding the indications of CIs and implanting more hearing-impaired individuals with significant residual hearing to enhance their quality of life. Evidence also suggests that individuals with more residual hearing stand to benefit more from CIs than individuals with less residual hearing in music and speech perception outcomes (Gfeller et al., 2006; Carlson et al., 2015; Chiossi and Hyppolito, 2017). Therefore, it becomes imperative to preserve residual hearing that can improve clinical outcomes of cochlear implantation (Eshraghi et al., 2017).

Although EIT-induced residual hearing loss is not fully understood, multiple mechanisms have been proposed that can lead to sensory cell damage. EIT can lead to a surge in reactive oxygen species (ROS) that can trigger caspase-3 dependent apoptotic pathways leading to HC death. Furthermore, the high ROS levels trigger an inflammatory response which leads to the production of proinflammatory cytokines such as tumor necrosis factor-alpha (TNF-α) in the cochlea (Wong and Ryan, 2015). TNF-α has been shown to activate the mitogen-activated protein kinase (MAPK) and the c-jun-N-terminal kinase (JNK) pathways that are also implicated in HC death (Wang et al., 2007). Studies have also implicated endolymphatic potential changes from EIT as well as depolarization of HC membranes that trigger apoptosis of HCs (Cohen-Salmon et al., 2002; Wangemann et al., 2004; Bas et al., 2016).

Pharmaceutical interventions targeting oxidative stress and apoptosis pathways hold great potential for developing therapeutic strategies for auditory disorders. In this context, bile salts such as tauroursodeoxycholic acid (TUDCA) have been shown to attenuate gentamicin-induced cochlear HC death in an *in vitro* study (Jia et al., 2018). Bile salts are amphipathic molecules that are derived from the breakdown of cholesterol and have steroidal properties (Davis et al., 2002). Studies have shown that bile salts have anti-apoptotic properties in a wide range of cell types including microglial, hepatic, and intestinal cells (Yanguas-Casás et al., 2017; Li P. et al., 2019). However, the efficacy of bile salts such as taurodeoxycholic acid (TDCA) in providing otoprotection against EIT has never been explored in previous investigations. TDCA is a bile acid taurine conjugate of deoxycholic acid and exists as a sodium salt. TDCA is produced in the liver and found mainly in the bile of mammals (Chiang, 2003). The differences between bile salts are minimal, as they differ only by positioning of hydroxyl groups in either position 3, 7, or 12 (St-Pierre et al., 2001). In this study, we determined the efficacy of TDCA in providing otoprotection against post-EIT induced HC death by employing a neonatal rat organ of Corti (OC) explants using a well-established *in vitro* EIT model.

## Materials and Methods

### Organ of Corti Explants

Three-day-old (P-3) Sprague–Dawley rats (Charles River Laboratories, Wilmington, MA, USA) were put on ice for 30 min as described in previous studies (Bas et al., 2012; Eshraghi et al., 2016). The OC explants were harvested and cultured in serum-free media containing Dulbecco’s modified Eagle’s medium (DMEM) with glucose (final conc. 6 g/l), N-1 supplement (1%), and penicillin G (500 μ/ml) supplements (all reagents from Sigma–Aldrich, St. Louis, MO, USA; Bas et al., 2012; Eshraghi et al., 2016; Tillinger et al., 2018). OC explants were incubated at 37°C in a 95% humidified atmosphere with 5% CO_2_. Explants were divided into three groups: (1) control group; (2) EIT alone group; (3) EIT and treated with different concentrations of TDCA (50 μM, 100 μM or 200 μM). For EIT, the cochleae were harvested from the P-3 Sprague–Dawley rats and a 0.28-mm diameter monofilament fishing line mimicking an electrode array was introduced through a small cochleostomy created next to the round window area. This procedure allows for insertion of between 110° and 150° as established in our previous studies (Bas et al., 2012; Eshraghi et al., 2016). The OC was harvested from each cochlea and cultured for 24 h with or without TDCA as described above. All the experiments were performed using OC explants. A total of 80 animals were used in this study. This animal study was conducted with the approval of the Animal Care and Use Committee of the University of Miami and fully complied with the NIH guidelines for the care and use of laboratory animals.

### FITC-Phalloidin Staining and Hair Cell (HC) Count

To visualize HCs, OC explants were subjected to FITC-phalloidin staining. Subsequently, the explants were cultured for 24 h and fixed in 4% paraformaldehyde (PFA; Electron Microscopy Sciences, Hatfield, PA, USA; Bas et al., 2012). Next, the explants were washed three times in phosphate-buffered saline (PBS; Sigma–Aldrich, St. Louis, MO, USA) and incubated in 5% normal goat serum (Sigma–Aldrich, St. Louis, MO, USA) and 1% Triton X-100 (Fluka, St. Louis, MO, USA) in PBS for 90 min at 25°C. Then the samples were washed three times with PBS and incubated with FITC-labeled phalloidin (Sigma–Aldrich, St. Louis, MO, USA) for 90 min at 25°C. Additional three washes were performed with PBS and then the samples were mounted with mounting medium having 4′,6-diamidino-2-phenylindole (DAPI) staining (Vector Laboratories, Burlingame, CA, USA), coverslipped, and viewed under a confocal Zeiss Axiovert 700 microscope (Carl Zeiss Microimaging, LLC, Thornwood, New York, NY, USA).

### Total Reactive Oxygen Species (ROS) Detection Quantification

ROS are associated with inflammatory processes as well as inducing apoptotic processes. Therefore, OC explants were subjected to CellROX staining as a marker of ROS. OCs were incubated with CellROX Deep Red reagent (5 μM, Thermo Fisher Scientific, Waltham, MA, USA) and incubated at 37°C for 30 min. The OC samples were then washed three times with PBS and fixed in 4% PFA for 24 h. Next, the fixed OC explants were washed three times with PBS and incubated in 5% normal goat serum (Sigma–Aldrich St. Louis, MO, USA) and 1% Triton X-100 (Fluka, St. Louis, MO, USA) in PBS for 90 min at 25°C. Additional three washes were performed with PBS and incubated with FITC-labeled phalloidin (Sigma–Aldrich, St. Louis, MO, USA) for 90 min at 25°C. Three additional washes were performed with PBS and then the samples were transferred to a glass slide with a DAPI-infused mounting medium (Vector Laboratories, Burlingame, CA, USA), coverslipped, and viewed under a confocal Zeiss Axiovert 700 microscope (Carl Zeiss Microimaging, LLC; Thornwood, New York, NY, USA). For CellROX deep red staining, the mean red signal intensity was measured as the average of nine regions of interest (ROI) and was normalized using the mean signal background intensity. The size and location of each ROI were consistent for all images. The mean signal intensity was measured and calculated using ImageJ version 1.52k software (Bethesda, MD, USA)^1^.

### Cleaved Caspase-3 and iNOS Immunolabeling

After fixation, the samples were washed three times with PBS and then incubated with one of two primary antibodies at a 1:200 dilution in PBS for 2 h at 25°C: (1) anti-cleaved caspase-3 (Asp175) rabbit polyclonal antibody (Cell Signaling Technology, Danvers, MA, USA); or (2) anti-iNOS antibody (BD Biosciences, San Jose, CA, USA). Subsequently, the samples were washed three times with PBS and incubated with secondary antibody Alexa 568-labeled goat anti-rabbit IgG (Thermo Fisher Scientific, Waltham, MA, USA) for 60 min at 25°C. The samples were then washed three times with PBS and incubated in 5% normal goat serum (Sigma–Aldrich, St. Louis, MO, USA) and 1% Triton X-100 (Fluka, St. Louis, MO, USA) in PBS for 90 min at 25°C. The samples were again washed three times with PBS and incubated with FITC-labeled phalloidin (Vector Laboratories, Burlingame, CA, USA) for 90 min at 25°C. Additional three washes were performed with PBS and then the samples were transferred to a glass slide with a DAPI-infused mounting medium (Vector Laboratories, Burlingame, CA, USA), coverslipped, and viewed under a confocal Zeiss Axiovert 700 microscope (Carl Zeiss Microimaging, LLC; Thornwood, New York, NY, USA). Both cleaved caspase-3 and iNOS staining were performed on OCs at 24 h post-EIT. For both the cleaved caspase-3 and iNOS staining, the mean red signal intensity was measured as the average of nine ROI and was normalized using the mean signal background intensity. The size and location of each ROI were consistent for all images. The mean signal intensity was measured and calculated using ImageJ version 1.52k software (Bethesda, MD, USA).

### JC-1 Mitochondrial Membrane Potential Quantification

Apoptosis is accompanied by the loss of Mitochondrial Membrane Potential (MMP). JC-1 (Thermo Fisher Scientific, Waltham, MA, USA) is a cationic dye that shows potential-dependent accumulation in the mitochondria. Increased accumulation of JC-1 in mitochondria shifts the fluorescence emission from green to red. This is due to the formation of J-aggregates at high concentrations which exhibit a red fluorescence pattern, whereas the monomer which exists at low concentrations has a green fluorescence. The ratio between red and green signal intensities can be measured to determine changes in the MMP. A decreased red/green ratio indicates a loss in MMP. For immunostaining, JC-1 was diluted in dimethyl sulfoxide (DMSO, Sigma–Aldrich, St. Louis, MO, USA) to a final concentration of 10 μg/ml. This working solution was then added to the culture media (1:250). Following incubation at 37°C with 5% CO_2_ for 2 h, explants were washed gently with PBS and mounted on a glass slide, cover-slipped, and viewed under a confocal Zeiss Axiovert 700 microscope (Carl Zeiss Microimaging, LLC; Thornwood, New York, NY, USA). ImageJ version 1.52k software (Bethesda, MD, USA) was used for processing and analyzing the images. Red (J-aggregates) and green (J-monomers) signal intensities were measured for each image using nine regions of interest (ROI) and the measurements were normalized using the mean signal background intensity. The average red/green intensity ratio was calculated for each image (Tillinger et al., 2018). The size and location of each ROI were consistent for all images.

### Statistical Analysis

A two-way analysis of variance (ANOVA) test was used followed by a Bonferroni post-test for multiple comparisons using SPSS software version 24.0 (New York, NY, USA). The data are expressed as mean values ± standard deviation (SD). A *p*-value of <0.05 was considered significant.

## Results

### TDCA Provides Otoprotection Against EIT Initiated Loss of HCs

**Figure 1:**
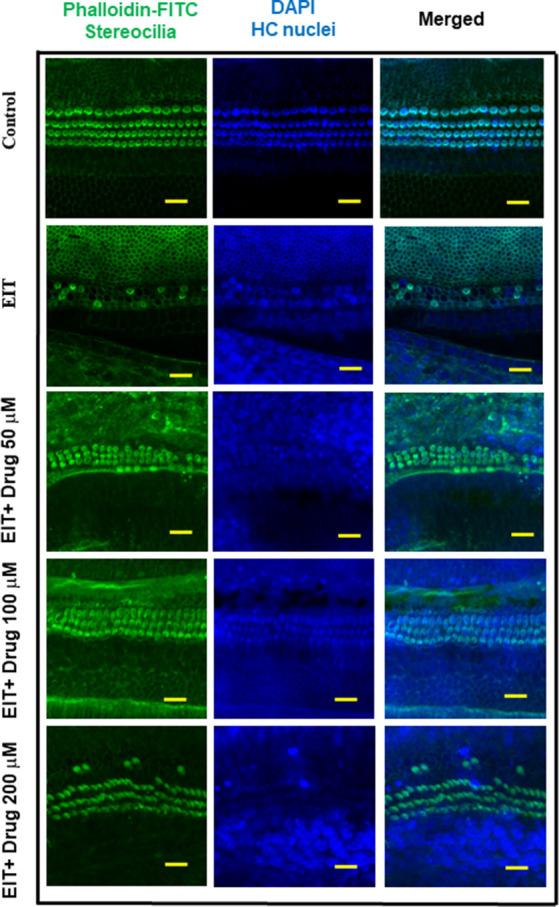
Taurodeoxycholic acid (TDCA) prevents hair cell (HC) loss from electrode insertion trauma (EIT). Representative images of FITC-phalloidin stained HCs in organ of Corti (OC) explants exposed to EIT alone or exposed to EIT and treated with different concentrations of TDCA. Results are representative of three independent experiments. *n* = 6 OCs per group. Scale bars: 10 μm.

**Figure 2:**
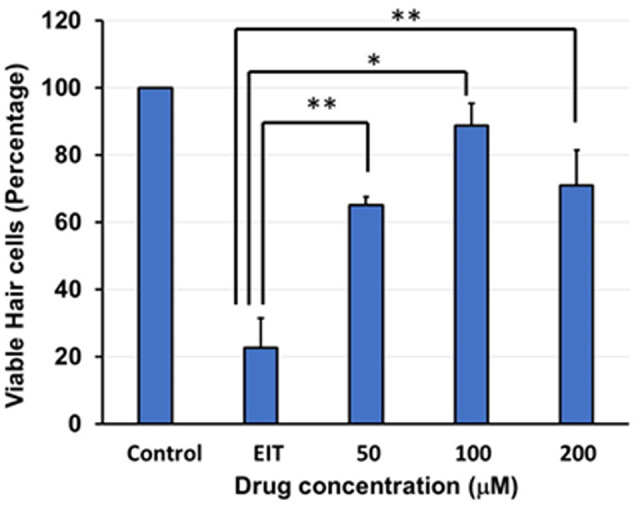
Quantification of HCs. Based on FITC-phalloidin staining, total HCs (outer and inner HCs) were counted and graphed. There was a decrease in total HC count in the OC explants subjected to EIT compared to the control group. TDCA was able to significantly prevent EIT induced sensory cell loss. Data are expressed as mean values ± standard deviation (SD) and are representative of three independent experiments. **P < 0.01 or *P < 0.001 compared to the EIT group. *n* = 6 OCs per group.

The OC explants from the P-3 rats were stained with FITC-phalloidin to visualize HCs. In the control group, OCs showed three rows of outer and one row of inner HCs. However, OCs subjected to EIT showed severe loss of HCs especially in the middle and basal turns (Figure 1). Treatment with TDCA significantly reduced the loss of HCs in response to EIT in a dose-dependent manner (*p* < 0.01). When EIT exposed OCs were treated with 50 μM of TDCA the percentage of total viable HCs was 50% that increased to 90% following treatment with 100 μM of TDCA (Figure 2). A further increase in the concentration to 200 μM provided no additional otoprotective effect in the preservation of HC loss. Therefore, 100 μM of TDCA was used for further experiments as this was observed to be the most effective concentration.

### TDCA Significantly Attenuates EIT Induced Oxidative Stress

**Figure 3:**
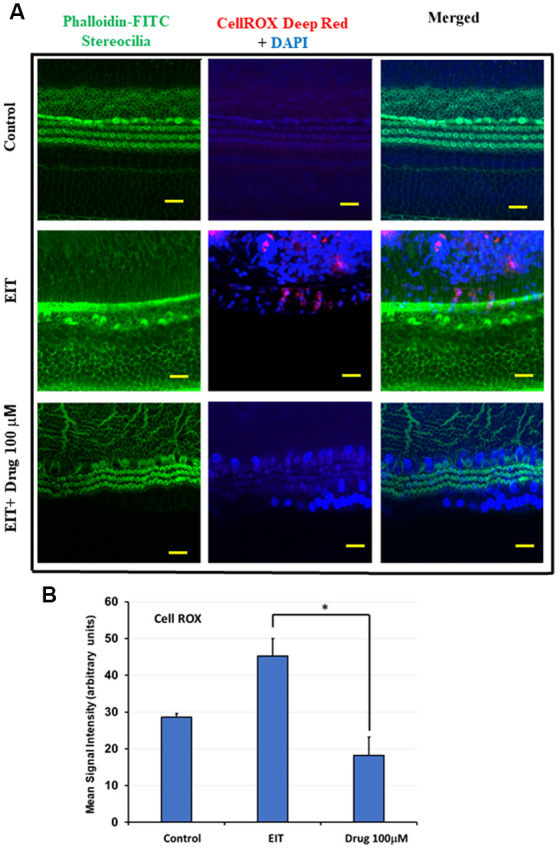
TDCA attenuates EIT induced oxidative stress in OC explants. **(A)** CellROX immunostaining in OC explants subjected to EIT or EIT and treated with TDCA. The first column shows the explant’s stereocilia bundles stained with FITC-phalloidin. Cell nuclei were stained with DAPI (blue color). **(B)** The mean signal intensity for CellROX was calculated using ImageJ software. Data are expressed as mean values ± SD and are representative of three independent experiments. *P < 0.001 compared to the EIT group. *n* = 6 OCs per group. Scale bars: 10 μm.

CellROX was used as a marker of oxidative stress. OC explants subjected to EIT showed strong immunolabeling for CellROX in the middle and basal turns (Figure 3A). Treatment with the identified compound caused a highly significant reduction in the levels of total ROS as indicated by a decrease in CellROX immunolabeling thereby effectively blocking the oxidative stress pathway. The mean signal intensity calculated with the ImageJ program was significantly reduced in EIT exposed OCs treated with TDCA (18.26 arbitrary units) compared to the EIT alone group (45.20 arbitrary units; *p* < 0.001; Figure 3B).

### Cleaved Caspase-3 Immunolabeling Is Reduced in OCs Treated With TDCA

**Figure 4:**
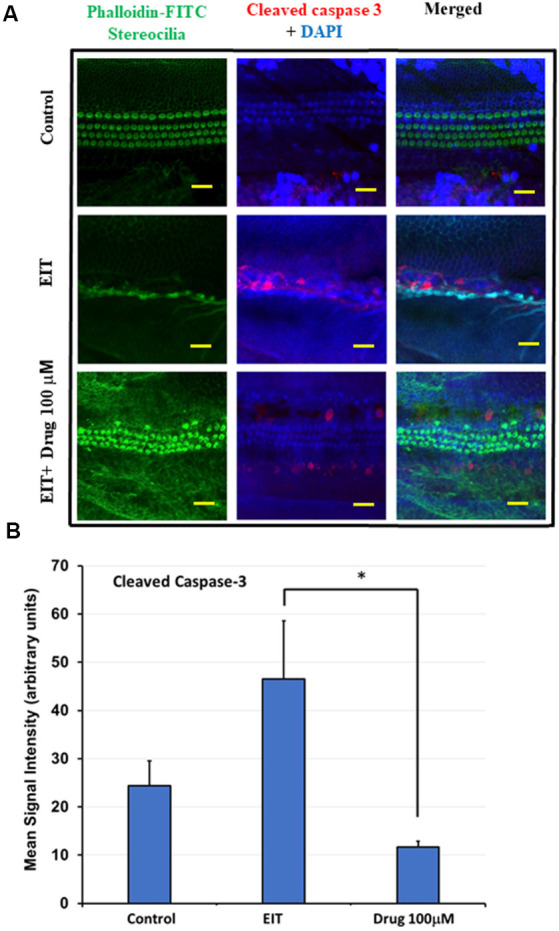
Cleaved caspase 3 immunostaining of OC explants treated with TDCA. **(A)** OC explants were subjected to cleaved caspase 3 immunostaining to determine apoptosis. The first column shows the explant’s stereocilia bundles stained with FITC-phalloidin. Cell nuclei were stained with DAPI (blue color). **(B)** Mean signal intensity for cleaved caspase 3 immunostaining was calculated using ImageJ software. Data are expressed as mean values ± SD and is representative of three independent experiments. *P < 0.001 compared to the EIT group. *n* = 6 OCs per group. Scale bars: 10 μm.

EIT activates the caspase-3 pathway leading to apoptosis of sensory cells. We observed significant cleaved caspase-3 immunolabeling in the middle and basal turns in EIT exposed OC explants compared to the control OC explants (Figure 4A). Treatment of OC explants with TDCA reduced EIT initiated cleaved caspase-3 immunolabeling compared to EIT alone. The mean signal intensity for the cleaved caspase-3 was significantly lower in TDCA treated EIT OC explants (46.49 arbitrary units) compared to the untreated EIT group (11.69 arbitrary units; *p* < 0.01; Figure 4B).

### iNOS Immunostaining of OC Explants Treated With TDCA

**Figure 5:**
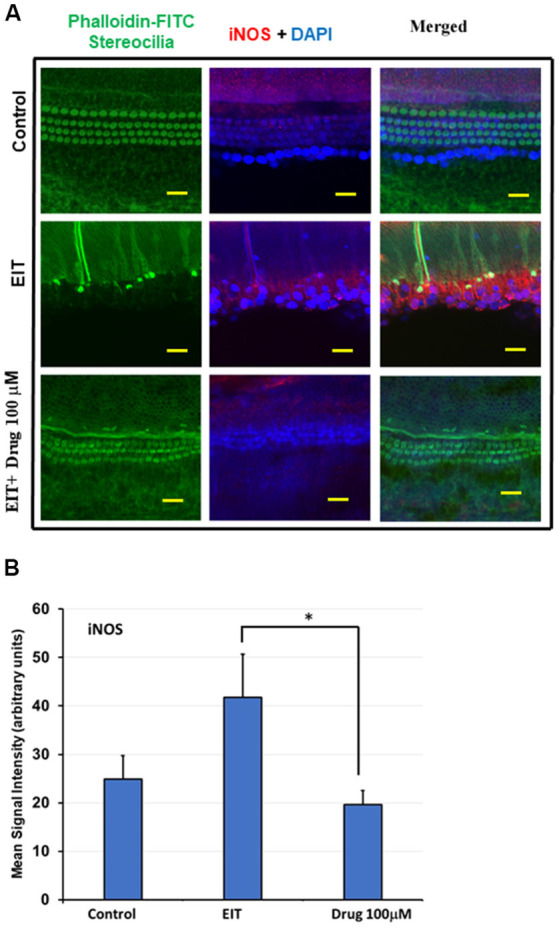
TDCA reduces inducible nitric oxide synthase (iNOS) immunolabeling in OC explants subjected to EIT. **(A)** Representative images of iNOS immunolabeling in OC explants. The first column shows the explant’s stereocilia bundles stained with FITC-phalloidin. Cell nuclei were stained with DAPI (blue color). **(B)** Mean signal intensity for iNOS immunostaining was calculated using ImageJ software. Data are expressed as mean values ± SD and are representative of three independent experiments. *P < 0.001 compared to the EIT group. *n* = 6 OCs per group. Scale bars: 10 μm.

Nitric oxide (NO) produced by iNOS can exert potent pro-inflammatory effects that can damage sensory cells. We observed significant iNOS immunolabeling in the middle and basal turns in the EIT exposed OC explants compared to the control OC explants (Figure 5A). Treatment of OC explants with the TDCA significantly reduced EIT induced iNOS immunolabeling. The mean signal intensity for iNOS immunolabeling was significantly reduced in EIT exposed OC explants treated with TDCA (19.60 arbitrary units) compared to untreated OC subjected to EIT alone (41.77 arbitrary units; *p* < 0.001; Figure 5B).

### Determination of Mitochondrial Membrane Potential (MMP) Using JC-1 Probe

**Figure 6:**
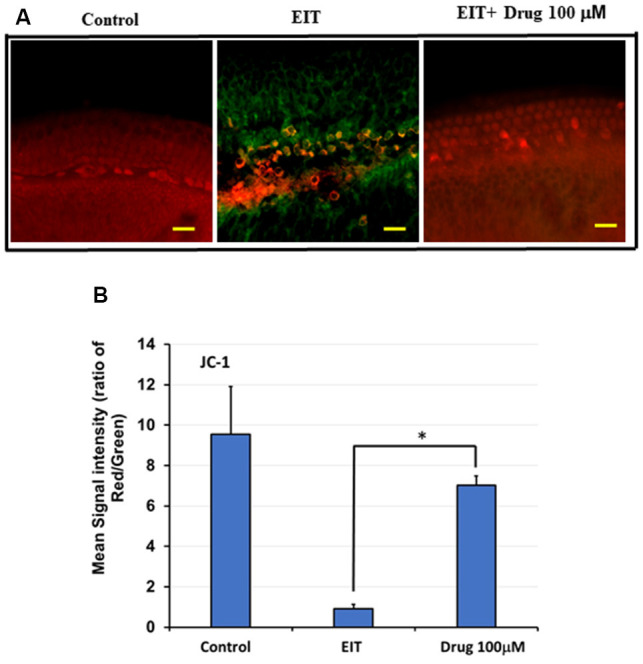
TDCA prevents EIT induced loss of mitochondrial membrane potential (MMP). **(A)** OC explants were stained with JC-1 probe to determine MMP. EIT exposed OCs showed red and green staining indicating loss of MMP whereas explants in control and TDCA treated group showed only red staining suggesting preservation of MMP. **(B)** The mean red/green staining signal intensity ratio was calculated using ImageJ software. Data are expressed as mean values ± SD and are representative of three independent experiments. *P < 0.001 compared to the EIT group. *n* = 6 OCs per group. Scale bars: 10 μm.

Immunostaining with the JC-1 probe was used as a measure of MMP (Δψm). Upon depolarization of this membrane, there is a fluorescence shift in staining from red to green. In the control group, we observed red staining indicating preservation of the MMP, whereas the EIT alone group showed red and green staining suggesting loss of MMP (Figure 6A). Treatment with TDCA was able to preserve this EIT-initiated loss of MMP as indicated by the presence of red staining and absence of green staining. The mean signal intensity ratio for the red to green signal was significantly reduced in the EIT group (0.92 arbitrary units) compared to the EIT OC explants treated with TDCA (7.01 arbitrary units; *p* < 0.001; Figure 6B). The results indicate that TDCA was able to maintain the MMP in the HCs.

## Discussion

The results of the present study suggest that TDCA is an otoprotective agent that can prevent sensory cell damage post-EIT. The importance of finding new otoprotective agents is signified in the fact that currently, despite advances in surgical techniques and improvements in CI technology, a significant number of implanted individuals still fail to preserve their residual hearing (Zanetti et al., 2015). The areas of exploration for hearing preservation and restoration utilize the modalities of gene therapy, genome editing, and stem-cell-derived therapies (Zou et al., 2015; Gao et al., 2018). Stem cell therapies using mesenchymal stem cells harvested from the human umbilical cord, induced pluripotent stem cells, and most recently, the utilization of patient’s stem cells, have emerged as promising avenues for combatting hearing loss (Mittal et al., 2017; Chorath et al., 2020). However, further studies are warranted to understand the underlying molecular mechanisms and develop avenues for effective delivery in human patients.

The stakes of preserving residual hearing have never been greater, as individuals with increased residual hearing are being implanted so that they can benefit from electro-acoustic stimulation whereby these individuals receive electric stimulation from the CI along with acoustic stimulation from intact HCs in the cochlea (Gantz et al., 2005; Gautschi-Mills et al., 2019). Additionally, implanted individuals with severe to profound hearing loss may also benefit from having preserved residual hearing when their device is turned off such as during sleeping and bathing. Preserving residual hearing adds a level of safety and boosts the self-confidence of implanted individuals. The loss of residual hearing that occurs after EIT has been attributed to a concerted effort by acute HC loss from direct trauma, subsequent peripheral HC loss from the activation of intrinsic and extrinsic pathways of programmed cell death (Dinh et al., 2015). The pharmacological interventions targeting oxidative stress, inflammatory, and apoptotic pathways hold great potential for developing novel treatment modalities for the preservation of residual hearing.

In the present study, we observed that TDCA provides otoprotection against EIT in our *in vitro* model. TDCA is a derivative of the bile salt deoxycholic acid. While there are very limited studies evaluating the efficacy of TDCA, a very close derivative, TUDCA, has shown to be a potent anti-inflammatory, anti-apoptotic, neuroprotective, and cardioprotective compound in a variety of diseases including primary biliary cirrhosis, enterocolitis, amyotrophic lateral sclerosis (ALS), and degeneration of cone cells in retinopathy (Elia et al., 2016; Li P. et al., 2019; Tao et al., 2019). One study showed TUDCA’s efficacy against necrotizing enterocolitis (NEC) which is a fatal disease affecting newborn babies. TUDCA reduced intestinal cell apoptosis by reducing caspase-3 and inflammatory cytokines such as IL-1β and IL-6 as well as blocking the endoplasmic reticulum stress pathway *via* activating the ATK pathway in an NEC mouse model (Li P. et al., 2019). ATK is a serine-threonine kinase that triggers pro-proliferation and growth pathways that antagonize apoptosis in the intestinal tract (Sun et al., 2017; Li P. et al., 2019). TUDCA is also being evaluated in human randomized controlled trials. One clinical trial enrolled 34 ALS patients that were randomized to placebo or treatment arms with TUDCA. The results showed that TUDCA had no increase in adverse events and may be a safe and effective treatment for ALS (Elia et al., 2016). There was a slower progression of disease in the TUDCA group than in the placebo arm. Additionally, studies have demonstrated the efficacy of TUDCA in inhibiting caspase-3 activation and apoptotic genes in Erl mouse cochleae. Erl mice have the *Cdh23*^erl/erl^ mutation which confers non-syndromic autosomal recessive deafness by altering the CDH23 protein in the upper part of the tip-link in HCs. These mice were a novel tool in assessing otoprotective drugs because of the time frame from the initiation of HC death to when complete deafness occurs (Hu et al., 2016). The experiments showed that the TUDCA-treated mice had significantly preserved as hearing determined by auditory brainstem response (ABR) and distortion product otoacoustic emissions (DPOAE) tests possibly due to the downregulation of caspase-3 and caspase-9 which was determined using western blot studies (Hu et al., 2016). TUDCA’s efficacy was also tested in gentamicin induced ototoxicity using both mouse primary OC explant cultures and an OC cell line, HEI-OC1. TUDCA treated samples had significant HC preservation and showed a decrease in iNOS expression (Jia et al., 2018). The results of our study are in agreement with these findings showing that TDCA has similar properties to TUDCA whereby it reduces caspase-3, iNOS, and ROS production and thus provides significant otoprotection.

Oxidative stress caused by the generation of ROS and reactive nitrogen intermediates (RNI) has been demonstrated to play a crucial role in sensory cell damage and loss of residual hearing (Jia et al., 2018). Extracellular ROS can cause cell death and necrosis by proteolytic degradation and peroxidation of the phospholipids in the cell membrane (Dinh et al., 2015). Intracellularly, ROS are formed by the mitochondria. If high levels of ROS accumulate then this can also lead to damage to the mitochondrial membrane and the release of pro-apoptotic factors into the cytoplasm (Dinh et al., 2015). In agreement with our previous studies, we observed intense CellROX staining in OC explants subjected to EIT, suggesting high levels of oxidative stress. TDCA was not only able to reduce the expression of ROS measured *via* the Cell ROX deep red staining, but also able to attenuate the RNI indicated by the reduction in iNOS immunolabeling. During inflammation, iNOS is upregulated and it produces NO which can be converted to peroxynitrite, an RNI. TDCA was able to decrease iNOS expression around the HCs and limit RNI production.

Besides oxidative stress, apoptosis in HCs involves either the intrinsic or extrinsic programmed cell death pathways. The extrinsic pathway involves a ligand such as TNF-α binding to a death receptor on the cell surface, triggering the activation of caspase 8 and eventual cleavage of caspase 3, initiating programmed cell death (Dinh et al., 2015; Eshraghi et al., 2015). Conversely, the intrinsic pathway begins with a stress signal that leads to an increase in the bax:bcl2 ratio in the mitochondria, which then triggers a channel opening in the outer mitochondrial membrane and the escape of pro-apoptotic molecules that trigger caspase-dependent (Cyt C) or independent pathways (Dinh et al., 2015; Eshraghi et al., 2015). Our previous studies have shown caspase-3 dependent sensory cell damage post-EIT (Eshraghi et al., 2015). In this study, we observed that TDCA was able to significantly reduce the expression of cleaved caspase-3, which can attenuate the activation of both the intrinsic and extrinsic pathways of programmed cell death. Interestingly, we observed that TDCA treated OC explants showed less cleaved caspase-3, iNOS, and CellROX deep red immunostaining than was observed in the control samples. One possible explanation for this can be attributed to the high potency of TDCA and its ability to downregulate gene expression of these inflammatory mediators; however, further studies are needed to confirm these findings.

The MMP is known to be a key indicator of cell health as it is essential for oxidative phosphorylation and for driving the import of essential proteins into the organelle (Friedman and Nunnari, 2014; Sakamuru et al., 2016). Loss of this membrane potential leads to the activation of signals that induce mitochondrial fragmentation and trigger subsequent cell death pathways *via* BAX activation as well as the release of ROS into the cytoplasm (Friedman and Nunnari, 2014). TDCA was able to protect the integrity of the mitochondrial membrane as indicated by the JC-1 probe which detects changes in MMP. This ascertains TDCA’s ability to attenuate the intrinsic pathway of apoptosis and prevent the release of pro-apoptotic molecules into the cytoplasm of HCs. Hence, the molecular mechanisms underlying TDCA otoprotection involve targeting oxidative stress, apoptotic, and inflammatory host pathways (Jia et al., 2013).

Bile acids and their potential for drug delivery have become a growing area of interest due to their unique inherent biochemical structures as well as their therapeutic effects in the body (Li and Chiang, 2020; Winston et al., 2020). Currently, bile acids are being explored as transport mechanisms for various drugs to increase targeted delivery and bioavailability (Dalpiaz et al., 2019; Kecman et al., 2020). These delivery strategies employ various bile acid-containing structures such as bilosomes, micelles, and bile-acid polymer nanocarriers (Pavlović et al., 2018; Albash et al., 2019; El Menshawe et al., 2020). All of these carriers serve as absorption enhancers, increase drug solubilization, and increase permeability into cells. Furthermore, bile salts can penetrate through the blood-brain barrier by modifying the tight junctions to allow for paracellular penetration or incorporation into the membrane bilayer (Lalić-Popović et al., 2013). In this case, since the bile acid, TDCA is itself the therapeutic agent it can be arranged into one of the aforementioned structures to facilitate its delivery to a specific part of the body such as the inner ear.

Liver damage and cholestasis due to bile acid toxicity have been a point of discussion limiting its therapeutic use. Studies have shown that the level of hydrophobicity in bile salts is the most important indicator of potential toxicity (Bernstein et al., 2011). However, bile acids conjugated to taurine, such as TDCA, have less hydrophobicity and thus less toxicity than glycine conjugated bile acids (Roda et al., 1990). Additionally, phosphatidylcholine, or hydrophilic bile acids such as Ursodeoxycholic acid (UDCA), can be added to micellar structures to reduce GI epithelium and membrane damage by the more hydrophobic bile acids (Barrios and Lichtenberger, 2000). With increasingly targeted mechanisms of delivery and chemical modifications to create less toxic bile acids such as TDCA, bile acids are becoming safer and more efficacious as potential drug therapies.

The limitations of our study include the lack of significant damage to the HCs in the apical part of the cochlea. This can be attributed to increased apical HC resilience or anatomical limitations posed by the *in vitro* model. Another limitation of our study is the use of only one technique (confocal microscopy). Future studies using multiple techniques such as flow cytometry will help in confirming the results of the present study.

In summary, our results suggest that TDCA provides an otoprotection against EIT. Further studies employing a preclinical guinea pig model of cochlear implantation established in our laboratory will help in confirming the results obtained from our *in vitro* model. Identification of novel otoprotective agents will help in developing novel treatment modalities to prevent loss of residual hearing post-cochlear implantation that will improve clinical outcomes in pursuit of improving the quality of life of implanted individuals and their families.

## Conclusion

Treatment of post-EIT organ of Corti explants with TDCA resulted in a significant reduction in HC loss when compared to EIT alone. Discovering novel otoprotective agents is an extremely important area of research as there is currently no indicated treatment for residual hearing loss due to EIT post-cochlear implantation. TDCA shows significant potential as an otoprotective agent post-EIT through its anti-oxidant, anti-inflammatory, and anti-apoptotic mechanisms of action.

## Data Availability Statement

All datasets generated for this study are included in the article.

## Ethics Statement

The animal study was reviewed and approved by the Animal Care and Use Committee of the University of Miami and fully complied with the NIH guidelines for the care and use of laboratory animals.

## Author Contributions

All authors listed have made a substantial, direct and intellectual contribution to the work, and approved it for publication.

## Conflict of Interest

AE is a consultant and received research funding from MED-EL Corporation. The remaining authors declare that the research was conducted in the absence of any commercial or financial relationships that could be construed as a potential conflict of interest.

## Footnotes

^1^https://imagej.nih.gov/ij
